# Can Neuroscience Assist Us in Constructing Better Patterns of Economic Decision-Making?

**DOI:** 10.3389/fnbeh.2017.00188

**Published:** 2017-10-10

**Authors:** George Lăzăroiu, Aurel Pera, Ramona O. Ștefănescu-Mihăilă, Nela Mircică, Octav Negurită

**Affiliations:** ^1^Department of Social-Human Sciences, Spiru Haret University, Bucharest, Romania; ^2^Department of Teacher Training, University of Craiova, Craiova, Romania; ^3^Department of Economic Sciences, Spiru Haret University, Bucharest, Romania; ^4^Department of Social-Human Sciences, Spiru Haret University, Bucharest, Romania; ^5^Department of Economic Sciences, Spiru Haret University, Constanta, Romania

**Keywords:** cognitive neurosciences, economic decision-making, brain investigations, value-based choice, neural design

## Abstract

We draw on outstanding research (Sanfey et al., 2006; McCabe, 2008; Bernheim, 2009; Camerer, 2013; Radu and McClure, 2013; Declerck and Boone, 2016) to substantiate that neuroeconomics covers the investigation of the biological microfoundations of economic cognition and economic conduct, attempts to prove that a superior grasp of how choices are made brings about superior expectations regarding which options are selected, preserves the strictness of economic analysis in defining value-based decision, and associates imaging techniques with economic pattern to explain how individuals decide on a strategy taking into account various possible choices. Neuroeconomics is adequately prepared to regulate the notion of how choices are determined by mental states. The position that will be elaborated in this article is that neuroeconomic patterns are enabled and enhanced in descriptive capacity by psychological outcomes and substantiated in biological processes. Advancement in neuroeconomics takes place when outcomes from distinct procedures are coherent with an ordinary mechanistic clarification of what generates choice, construed by a computational pattern. We will develop this point further by proving that economics improves the concerted effort of neuroeconomics by using its observations in the various results that may stem from the planned and market interplays of diverse participants, and via a series of accurate, explicit, mathematical patterns to construe such interplays and results. Neuroeconomics experiments employ a mixture of brain imaging/stimulation tests advanced in the cognitive neurosciences and microeconomic systems/game theory tests advanced in the economic sciences. Our analyses indicate that neuroeconomics aims to employ the supplementary input gained from brain investigations, associated with the decision maker’s selection, with the purpose of better grasping the cogitation process and to utilize the outcomes to enhance economic patterns.

## Introduction

Neuroeconomics is the investigation of how individuals make value-based choices and how the latter are conveyed neurally, cognitively, and behaviorally (the most advancement has been carried out in grasping the sense of rewarding stimuli and how the brain discerns to ascribe them value). A complete account of value-based decision (Ahmed, 2016) requires various synchronized degrees of investigation. Neuroeconomics improves the established economic proposal to shaping decision in two significant manners: (i) integrating inquiry in psychology, breaches of sound judgment are identified and admitted as pervasive (individuals often depend on preconceptions and heuristics that are furthered and influenced by previous experience); and (ii) systems neuroscience has proved that the brain operates in a corresponding, distributed way in order that input is handled synchronously by diverse specially designed systems. Neuroeconomics preserves the strictness of economic analysis in defining value-based decision (Mihăilă, 2016a) and admits that decision-making is driven in an evolutionary way and not inevitably optimal. Neuroeconomic patterns are enabled and enhanced in descriptive capacity by psychological outcomes and substantiated in biological processes (Radu and McClure, 2013).

## Should Economics Embrace Neuroscience?

Neuroeconomics covers the investigation of the biological microfoundations of economic cognition and economic conduct, mirroring a reductionist method to social science that is based on two assumptions: (i) that clarifying systems for construing human choice behavior may be advanced at neuroscientific, psychological and economic layers of exploration; and (ii) that there may be coherent and comprehensible mappings among such stages of elucidation. As such mappings are determined, a flow of algorithmic limitations from neuroscience may become feasible to economists. Equivalently, normative patterns and empirical behavioral ones from economics (Laudan et al., 2016) may be instrumental in restricting neurobiological patterns. Investigations of single neurons provide indications of a well-defined mapping between economic principles and brain function (Camerer et al., 2016). As the discipline of choice, economics is hindered by insufficient resources and organizational structure. Neuroeconomics has the same chief objectives as microeconomics: to grasp what generates choices, and the welfare features of choice, offering confirmation of circumstances in which utility maximization operates satisfactorily in simple binary choice (Nica et al., 2016b) or gains from the establishment of behavioral paradigms, thus being adequately prepared to regulate the notion of how choices are determined by mental states. The innovative aim is connecting mathematical paradigms and perceivable conduct to mechanistic facets of neural circuitry. Different systems drive choice behavior: Pavlovian and instrumental training of state-value and response-value relationships, overlearned routines and pattern-directed value that entails cerebration. Such systems may be dissimilar economically from rational choice. The raised superiority and diversity of neural measures may cause technological replacement (Lucas, 2016), from conjecture regarding undetectable cognitive variables contingent entirely on observed choice, to precise assessment of such variables and choices (Camerer, 2013).

The model of neural stimulation triggering any conduct is brought about by a brain and evolutionary preserved neuropeptide systems unceasingly shaped to match various environmental constraints. Brain plasticity enables group action and acquirements, fine-tuning decision making consistent with local circumstances (Zapparoli et al., 2017): the brain is strengthened to integrate heterogeneous sets of information simultaneously, separating out conducts that turn out to generate typically the uppermost fitness gain. At the next level, prosocial decisions are induced by the expectation of an economically advantageous or emotionally gratifying reward. The importance associated with teamwork is set up in the brain’s reward system, which acquires information from a cognitive control system estimating the advantages to the person (Layard, 2016), and a social cognition one, which is responsive to intricate social input concerning the reciprocal purposes of other individuals. Which system predominates in the decision-making process is determined by the local circumstances (Terry, 2016), i.e., the occurrence or unavailability of motivators and the sense of trustworthiness of other individuals, and established predispositions or options to act either prosocially or egoistically. Prosocial decision making harmonizes both economically and socially coherent reasons. Pursuing the economically coherent path to teamwork, individuals feel stimulated to follow self-interest, but collaborate voluntarily when self-interest corresponds to shared interest. Pursuing the socially coherent path, individuals aspire to group inclusion, and teamwork is a successful manner to fortify belonging, establish social networks (Tomasi et al., 2017) and circumvent exclusion (Declerck and Boone, 2016).

A sound process-oriented neural assessment of any decision axiom necessitates a systematic comprehension of the connections between the attributes of choice mappings, the features of decision designs, and the characteristics of neural frameworks. Indications relevant to the way in which individuals perceive and handle factual information may clarify the degree to which certain decisions are successfully learned, and consequently suitable as benchmarks for normative investigation. Notwithstanding having access to certain evidence, a decision maker may be unsatisfactorily responsive, be unsuccessful in bringing to mind significant pieces of information that associate decisions with outcomes (Kocsel et al., 2017), estimate the end results of his decisions erroneously, or acquire information from his previous actions more gradually that the achievable evidence would allow. By investigating the neurobiology of awareness, memory, prediction and knowledge, particular circumstances under which individuals are either actually misled or essentially less well-informed may be identified than they would be by accurately handling all the obtainable factual information. Neuroeconomic approaches may assist individuals in forecasting flawlessly the decisions that other persons would make from a particular series of expectations by assessing their neural responses to those likelihoods, even when no option is provided (Bernheim, 2009).

The theory of economic conduct in relation to neuronal computation is hard to conceive. The suitability of neuroeconomics to economics analysis is associated with the wider issue of the effectiveness of the experimental approach in economics and to individuals’ awareness of how the brain operates, and how to assess that. By advancing the research of decision making to the brain, neuroeconomics focuses more on choices (Popescu et al., 2016a), encoding of evidence, and cognition, making economic theory more prognosticative. The objective-directed brain is constructed to employ strengthening learning schemes to optimally utilize the impersonal regulations established by an organization and to bring into play cooperation to initiate personal arrangements (Popescu Ljungholm, 2016) that enable individuals to dodge and alter the regulations of their organizations. In grasping human exchange conduct individuals have to comprehend how this inconsistency gets resolved in their brains and thus how the inducements and evidence generated by their organizations resolves this inconsistency with the purpose of harmonizing the employment of personal and impersonal network in a formal society. Individuals confront numerous opportunity cost transactions in their ordinary undertakings. Such arrangements are designed as a scheme that represents information sets into operations (McCabe, 2008).

## Economic, Psychological and Neurobiological Patterns of Human Choice Behavior

The presence of various neural systems of assessment and choice is active at the center of the disagreement between fundamentalist decision theory and neuroeconomics: fundamentalists believe that nothing but the restrictive utilization of revealed-preferences modeling to attain this aim is crucial for economics (Nica, 2016), whereas neuroeconomists think that as choices result from various systems, grasping choice thoroughly necessitates ultimately comprehending such systems (the objective of economics is to envisage choices and comparative statics effects). Neuroeconomics may progressively inspire an awareness that biological mechanisms are significant ingredients of separate choice, influence particular instances of how to shape such mechanisms conventionally in a knowledgeable manner (Bolton, 2016), present unexpected causal impacts on choice, and supply novel data furthering positions that formulate detailed assertions concerning both neural activity and choice. Advancement in neuroeconomics takes place when outcomes from distinct procedures are coherent with an ordinary mechanistic clarification of what generates choice, construed by a computational pattern (Camerer, 2013).

The brain is equipped for both an economic and a social coherence. Economically and socially coherent preferences are entrenched in distinct neural networks that function harmoniously and separately regulate decision making. Prosocial decisions may be interpreted as driven preferences (Popescu et al., 2016b) that generate either economically or socially beneficial rewards. Such choices are based on the occurrence of extrinsic rewards that line up self-interest with shared interest and/or rely on indications that decrease the likelihood of exploitation. The neural networks covering *reward processing*, *cognitive control* and *social cognition* are the brain systems reliably employed when individuals confront confusing situations that entail teamwork, being connected in such a manner that an (un)cooperative choice is the consequence of the modulatory effects of the latter systems on the former one. Persons who are inherently motivated to behave prosocially, experiencing the emotional and shared advantages of reciprocal collaboration, may alter this conduct when they perceive it is not any longer adjustable (Declerck and Boone, 2016).

Economics improves the concerted effort of neuroeconomics by using its observations in the various results that may stem from the planned and market interplays of diverse participants, and via a series of accurate, explicit, mathematical patterns to construe such interplays and results. The feature of economics that might be extremely constructive to neuroscientists is its taking over of an integrated theoretical scheme for comprehending human conduct. The latter may be viewed as selecting options with the objective of augmenting utility and is not the outcome of an individual mechanism (Xia et al., 2017), but to a certain extent indicates the interplay of distinct specialized subsystems. Even though these systems generally combine synergistically to regulate conduct, now and then they oppose, generating various dispositions with regard to the same input. An important explanation of such peculiarities of conduct that have been employed to question the customary economic pattern (Popescu et al., 2016b) may be that the choices do not arise from an integral process, but in a certain degree from interplays between discernible series of processes (Sanfey et al., 2006).

Neuroeconomics attempts to prove that a superior grasp of how choices are made brings about superior expectations regarding which options are selected. To recognize the causal consequence of a person’s selection on another’s option (Klosse and Muysken, 2016), a variable that straightforwardly impacts the choice of a person is needed. Neural predispositions possibly have such property. Neuroeconomics may ascertain confines on the consistency of neurally imputed choices (e.g., estimates may be less precise when a person is presented with a considerable amount of options). Under some circumstances, neural responses might pursue hypothetical choices (Hurd, 2016) more precisely than actual ones. Imputations might be less consistent when the selection is uncommon, and/or responsive to presentation when the options are heterogeneous. Nearly all economic approaches of decision making bring up suppositions about choice models, and are skeptical regarding the character of decision processes (Bernheim, 2009).

Brains are appropriately modeled for objective-directed conduct employing strengthening learning mechanisms. Individuals devise institutions (e.g., money), to further their objective-directed conducts (Flynn, 2016), and such institutions consecutively get created in the brain. Decision-making necessitates the synchronized undertaking of motivational, emotional and cognitive connections to formulate, perceive and evaluate possible choices, get involved and acquire information from suitable feedback. The brain conserves scarce neuronal resources. In end results that generate both direct functional incentives (e.g., soda), and indirect functional incentives (e.g., money), the motivational system converts an expected incentive value for the end result with the purpose of bringing up an undertaking. When the end result is related to the undertaking, the motivational system estimates an accomplished value (Libey and Fetz, 2017), weighs it against the expected value, and when there is a dissimilarity the brain requires scarce resources to amend its scheme. The brain can intrinsically assess an institutional or cultural concept (e.g., money), in regard to its end use, and subsequently employ this appraisal to stimulate learning (McCabe, 2008).

## The Neural Design Encompassed in Decision Making and the Modeling of Economic Choice Structures

Neuroscientific data supply relevant information regarding the circumstances and mechanisms that affect economic conduct. A first-rate method employed for producing such data is functional magnetic resonance imaging (fMRI) that offers significant information concerning the neural processes comprised in valuation and decision making. An important advance to experimental configuration and examination of fMRI data is the utilization of subtractive logic in order to determine confined areas of neural activity related to mechanisms of interest. More elaborate procedures involve correlation-based investigations to establish interplays among brain areas (Brown, 2016), and the utilization of multivariate approaches to detect distributed models of activity related to mental states and mechanisms. Such enhancements may make it feasible to determine and pursue, with growing capacity and accuracy, the sophisticated brain computations contained in human mental function, comprising ones that are essential to economic decision making. Neuroeconomic investigations of risky choice constitute a sound synthesis of parametric modeling present in economics and decision theory (Mihăilă, 2016b) and wide-ranging kinds of neural assessment borrowed from neuroscience. Risk and reward are consistently converted in insula (risk) and striatum and medial orbitofrontal cortex (OFC, reward; Camerer et al., 2016).

Temperamental dispositions in conjunction with experience-based dissimilarities in social acquirements may cause substantial alterations in values that are pursued by particular activation models of the brain’s reward system. Values represent a knowledge instrument that assist individuals in plotting a route through the social realm (Popescu et al., 2016b): they establish which substantial information persons are more probably to focus on, affecting the extent to which networks covering cognitive control or social cognition are employed in the decision-making process. Each person improves his own neural signature that furthers or impedes teamwork. Economically or socially coherent cooperation heuristics within the confines of a certain social group are likely to influence decision making to the advantage of same-group members (Machan, 2016a), but when that group is pressurized such biased conduct might develop into extreme forms. Prevailing over the adverse secondary effects of prosociality depends on the neural network of cognitive control related to economically coherent decision making. The notion of rationality, as regards cooperative conduct (Machan, 2016b), should take into account a person’s intrinsic values. In recurrent social interplays, the advantages of teamwork, whether via concerted effort, accruing profits, or via (in)direct mutual benefit, can be determined, and thus prosocial conduct is economically rational (Declerck and Boone, 2016).

Self-regulating processes are swift and well-organized, may frequently be performed at the same time, but are greatly focused for domain-specific undertakings and thus somewhat strict, signifying the activity of extremely over-trained mechanisms. Individuals similarly can supervise processing triggering superior cognitive faculties. Furthermore, controlled processes are tremendously manageable (Nica, 2017), and consequently able to reinforce a broad diversity of objectives, but are rather time-consuming to participate and depend on confined capacity mechanisms, maintaining only a negligible amount of undertakings simultaneously. The performances comprised in controlled processes are frequently available to inward-looking, unambiguous account (Weede, 2016), while ones encompassed in self-regulating processes are typically even less so. Economics may be instrumental in comprehending the active mechanisms by which the brain synchronizes its various systems to carry out novel, intricate tasks. Both the brain and a company may be regarded as multifaceted systems converting inputs into outputs (Friedman et al., 2016), requiring the interplay of manifold, greatly similar, participants, which are specially designed to accomplish certain functions. In companies, units frequently develop into departments that bring about activities such as research, marketing, etc. The brain cumulates systems focused on distinct functions, and as in a company, the latter may be spatially isolated in the brain (Fujita et al., 2017), relying on the processing demands of the particular roles and their interplays. A hierarchical structure can be identified in both brains and companies, both depending upon executive systems that assess the relative relevance of undertakings and choose how to catalyze specially designed capabilities to carry them out (Sanfey et al., 2006).

Neuroeconomics experiments employ a mixture of brain imaging/stimulation tests advanced in the cognitive neurosciences and microeconomic systems/game theory tests advanced in the economic sciences. Economists assess information, while neuroscientists evaluate brain activity, the latter generating the information supplying a supplementary source of evidence to typify subjects. Economists attempt to conjecture subjects’ schemes from the information generated (Kantarelis, 2016), while neuroscientists endeavor to surmise the neuronal processes that brought into being the perceived neural activations (schemes are images of the role that the processes carry out). Economists design the perceived schemes in relation to equilibrium conditions, whereas neuroscientists shape the neuronal processes with reference to the estimations they operate (equilibrium and data processing are systematized by the general criteria of optimization; McCabe, 2008).

## Conclusion

Neuroscience (and more specifically neuroeconomics) can not only enhance our understanding of the decision making processes as we see them through the lenses of the classical economic theoretical framework, but can, at the same time, also challenge the same framework (Hunt et al., 2015; Hunt and Hayden, 2017). Neuroeconomics supplies an empirically directed, computationally thorough fabric for theorizing addiction as an instance of biased reward returning, and uses the parameterized choice models of conventional and behavioral economics (Radu and McClure, 2013). Neuroeconomics aims to employ the supplementary input gained from brain investigations, associated with the decision maker’s selection, with the purpose of better grasping the cogitation process (Popescu, 2016) and to utilize the outcomes to enhance economic patterns. Neuroeconomics’ proposal may be constructive in two manners: (i) once individuals have augmented economic patterns of bounded rationality (Nica et al., 2016a), procedures should not be devised without precise knowledge but to apply patterns contingent on their insight of the mind (consistent neuroeconomics data may be useful by supplying individuals with information regarding how pervasive the employment is of a certain decision-making practice); and (ii) brain investigations may assist in categorizing kinds of people who share types of conduct for a broad series of choice scenarios: individuals would be stimulated to build up patterns in which the allocation of kinds is a primitive of the pattern. Employing such patterns individuals may obtain more convincing analytical outcomes (Rubinstein, 2008). Neuroeconomics associates imaging techniques with economic pattern to explain how individuals decide on a strategy (Di Domenico and Ryan, 2017) taking into account various possible choices (it attempts to grasp the physiological and neural processes determining decision making). The intrinsic mechanisms of assessment is a reward-seeking brain whose role is to harmonize environmental input with personal values to reach a choice that complements one’s estimated separate likelihood (Declerck and Boone, 2016).

## Author Contributions

All authors listed have made a substantial, direct and intellectual contribution to the work, and approved it for publication.

## Conflict of Interest Statement

The authors declare that the research was conducted in the absence of any commercial or financial relationships that could be construed as a potential conflict of interest.
